# Investigating the prevalence of *Salmonella* in dogs within the Midlands region of the United Kingdom

**DOI:** 10.1186/s12917-015-0553-z

**Published:** 2015-09-17

**Authors:** Preena Lowden, Corrin Wallis, Nancy Gee, Anthony Hilton

**Affiliations:** Life and Health Sciences, Aston University, Birmingham, B4 7ET UK; WALTHAM Centre for Pet Nutrition, Waltham-on-the-Wolds, Leicestershire, LE14 4RB UK

**Keywords:** *Salmonella*, Zoonosis, Dog, Epidemiology, Prevalence

## Abstract

**Background:**

The intimate relationship between dogs and their owners has the potential to increase the risk of human exposure to bacterial pathogens. Over the past 40 years, there have been several reports on transmission of salmonellae from dogs to humans. This study therefore aimed to determine the prevalence of *Salmonella* in the faeces of dogs from the Midlands region of the United Kingdom to assess exposure risk and potential for zoonotic transmission.

**Results:**

A total of 436 apparently healthy dogs without diarrhoea from households (*n* = 126), rescue centres (*n* = 96), boarding kennels (*n* = 43), retired greyhound kennels (*n* = 39) and a pet nutrition facility (*n* = 132) were investigated for *Salmonella* shedding. Faecal samples were processed by an enrichment culture based method. The faeces from one dog (0.23 %; 95 % confidence limit 0.006 %, 1.27 %) was positive for *Salmonella.* The species was *S. enterica* subspecies *arizonae*.

**Conclusion:**

This study showed that the prevalence of *Salmonella* from faeces from apparently healthy dogs from a variety of housing conditions is low; however, Salmonella shedding was still identified.

## Background

*Salmonella* is the aetiological agent of both human and animal salmonellosis, a very common and widely spread enteric disease. Over 7,500 human cases of *Salmonella* infections were reported in the United Kingdom (UK) in 2013 [1]. It has been estimated that 55 % to 95 % of human salmonellosis cases are foodborne and that approximately 9 % are attributable to direct animal contact [2–5]. With respect to salmonellosis cases that may be attributable to pets the estimates are lower at approximately 3 % [6]. According to the reports of physicians and veterinarians, salmonellosis rates as the second most serious zoonotic disease after toxoplasmosis. It poses most risk to children, the elderly and immunocompromised humans [7, 8].

In 2014, it was estimated that there were approximately nine million dogs in the UK [9]. Dog ownership is associated with many benefits for people including companionship and physiological and psychological health [10–16]. In order to maximize these benefits it is also helpful to understand any risks to public health from zoonosis [17]. Over the past 40 years there have been several reports on transmission of salmonellae from dogs to humans [18, 19]. Today, dogs in Western Europe primarily live indoors, share living spaces with their owners, and assume integral roles as companions, family members, or service animals [20, 21], and this more intimate relationship between dogs and their owners has the potential to increase the risk of human exposure to *Salmonella*.

Dogs have been reported to harbour and shed *Salmonella* subclinically. High numbers of the microorganism can reside in the intestines and mesenteric lymph nodes without clinical signs [22] making an estimate of the prevalence of salmonellae in dogs in the community difficult to establish. The prevalence of subclinical carriage of *Salmonella* in clinically healthy dogs varies greatly among individual countries and has been reported to range from 0 to 44 % [23, 24]. However, recent studies on the prevalence of *Salmonella* in different populations of dogs within the UK are lacking [25–27]. The objective of this study was therefore to estimate, and potentially compare, subclinical carriage of *Salmonella* in dogs housed in a variety of settings, including household, pet nutrition facility and kennelled dogs from the Midlands region of the UK, to determine their potential as a source of *Salmonella* infection to humans.

## Results

A total of 436 faecal samples were collected from different populations of dogs (see Table 1). Descriptive information on the sample of dogs in the study, breed groups (according to The Kennel Club Organisation [28]), age, sex, weight and diet, are provided in Table 2. The majority of the dogs were from the gundog group with hound, pastoral and utility being the next most represented. This representation of breeds is similar to that reported by The Kennel Club Organisation as among the most popular in the UK based on current registrations [28]. The dogs ranged in age from 0.3 to 16 years and weights ranged from 2 to 48 kg. There were 227 male and 209 female dogs in the study and the majority of females were intact (64 %) and the majority of males were neutered (74 %). A total of 30 dogs (6.9 %) were fed a home prepared diet and the remaining dogs (93.1 %) were fed a commercial diet. Only two dogs, representatives of the hound and gun dog breed groups, were fed a home-prepared raw meat diet.

**Table 1:** Number of faecal samples analysed and the number positive for *Salmonella* from different dog populations

Population	Total number of dogs	Number of dogs *Salmonella* positive	*Salmonella* serotype
Households	126	1	*S. arizonae*
Rescue centres	96	0	-
Boarding kennels	43	0	-
Retired greyhounds	39	0	-
Pet nutrition facility	132	0	-

**Table 2:** Breed group, age, gender, weight, and diets of dogs included in the study

Breed group	Number of dogs	Age (years)		Gender (no. of dogs)				Weight (Kg)		Diet					
										Commercial			Home prepared		C&H
		Range	Median	F	Fn	M	Mn	Range	Median	Wet	Dry	Mixed	Cooked	Raw	
Gundog	155	0.5-14	5	48	27	21	59	6-39	25	18	66	60	9	1	1
Hound	68	2-15	4	13	16	13	26	4-32	22	1	44	22	-	1	-
Pastoral	68	1-16	6	23	8	4	33	13-38	16	7	16	36	9	-	-
Terrier	39	0.4-12	3	18	0	7	14	3-28.5	15.5	2	13	24	-	-	-
Toy	9	1-6	3	4	0	4	1	2-7	4	-	-	8	1	-	-
Utility	64	0.3-13.6	3	16	15	6	27	8-36.3	12	12	29	23			
Working	14	3-8.1	5	5	2	2	5	32-48	32		1	8	3	-	2
Cross Breeds	19	1.6-14	6	7	7	2	3	13-39.1	22	1	2	13	-	-	3
Total	436	0.3-16	4.5	134	75	59	168	2-48	19	41	171	194	22	2	6

*F* female, *M* male, *Fn* female neutered, *Mn* male neutered, *C&H* commercial and home prepared

Using an enrichment culture method along with three selective agars, only one of the 436 faecal samples (0.23 %, 95 % confidence interval 0.006, 1.27) was positive for *Salmonella* (see Table 1)*.* The species was *S. enterica* subspecies *arizonae.* The *Salmonella* positive dog was a female terrier breed from a household population, aged four and weighing 7 kg. The questionnaire revealed that the dog was fed a commercial diet (mix of wet and dry) and had a tendency to scavenge when outdoors in the garden. Subsequent repeat samples taken from this dog over three consecutive days, one week after the first sample, were negative for the presence of *Salmonella.*

When using direct culturing methods, none of the faecal samples in the study were positive for *Salmonella* shedding. When both direct and enrichment culturing methods were used, samples taken from kennel groups of dogs (rescue centres, boarding kennels, retired greyhounds and pet nutrition facility) were all negative for *Salmonella* shedding. Given the low prevalence of *Salmonella,* restricted to only one of the dog populations, further statistical comparisons between the household, pet nutrition facility and kennelled dogs was not undertaken as it was considered to be inconsequential.

## Discussion

Apparently healthy dogs can harbour *Salmonella* and might thereby serve as a potential source of human infection with implications for public health. Almost a quarter of all UK households are home to at least one dog [9] and it is therefore important that we understand the risk of transmission of zoonotic infections. This study showed that the prevalence of *Salmonella* in dogs located in the Midlands region of the UK is low (0.23 %; 95 % confidence interval 0.006 %, 1.27).

Other studies have estimated the prevalence of subclinical carriage of *Salmonella* in clinically healthy dogs to range from 0 to 44 % [23, 24]. The results of the current study are in line with the lower estimates, but are inconsistent with the larger estimates. The prevalence of subclinical shedding of *Salmonella* in normal apparently healthy household dogs has been reported for a number of different countries. A study of 150 dogs from Hawke’s Bay, New Zealand showed an absence of subclinical carriage [29]. By contrast, a study of 251 dogs visiting parks in three cities in south-western Ontario, Canada reported *Salmonella* in 1.2 % of the dogs [30]. Other studies have reported slightly higher prevalence. Rectal swabs collected from 437 household in northern Taiwan revealed that 2.1 % (9 dogs) were positive for *Salmonella spp.* [31]. A study of 1391 dogs across Trinidad reported a prevalence of 3.6 % [32] and investigations undertaken in Tehran, Iran and Florida, USA reported even higher prevalences of 4.4 % (21 of 474 dogs) and 15 % (*n* = 1,626) respectively [33, 34]. This considerable geographical variation in the prevalence of *Salmonella* serotypes reported in dogs could be due to the sample size, year of sampling, sampling strategies, and isolation methods performed, but may also be due to cultural differences in feeding or hygiene practices or favorable climate conditions for bacterial growth and survival.

The prevalence of *Salmonella* in dogs as reported in the literature is also highly variable depending on the immediate environment in which the animals live. For example, *Salmonella* isolation rates from stray dogs have been reported to be significantly higher than those from household dogs [31]. For this reason, in the current study faecal samples were collected and analysed for the presence of salmonellae from dogs housed in a variety of environments: households, rescue centres, boarding kennels, retired greyhound kennels and a pet nutrition facility. It is noteworthy that in this study all kennelled dogs tested negative for the presence of *Salmonella,* although other studies have reported a higher prevalence of *Salmonella* shedding in kennelled dogs. Rectal swabs from kennel dogs in Tehran, Iran indicated that 28 out of 181 (15.5 %) were positive for *Salmonella* [33]. In a shelter in Bursa, Turkey 11 % of dogs tested positive for *Salmonella* [35], and in Japan, 5.9 % of stray or unwanted apparently healthy dogs were positive for the presence of salmonellae in their intestinal contents [36]. In northern Taiwan rectal swabs collected from 491 stray dogs in a municipal animal shelter found 6.3 % (31 dogs) dogs were positive for salmonellae [31]. However, other studies involving strays or kennelled dogs show much lower rates of carriage that are more in line with the findings presented here. Ojo [37] failed to detect salmonellae in the intestinal contents of stray dogs (*n* = 100) in Trinidad, West Indies, and an analysis of rectal swabs collected from kennelled dogs in Istanbul, Turkey found only one out of 100 (1 %) to be positive for *Salmonella* [38].

Shedding of *Salmonella* in faeces is also known to be common among the racing greyhound population. Racing greyhounds in the USA have been shown to have high rates of subclinical shedding of *Salmonella* at 44 % [39], and a more recent study reported *Salmonella* in faeces from 11 % of asymptomatic greyhounds [40]. The high prevalences of *Salmonella* that are typically reported in greyhounds may be traceable to the high-protein raw meat diet provided for racing. A high prevalence of *Salmonella* in these raw meat diets has been reported, and identical enterotypes have been found in the faeces of dogs consuming the food, confirming that the diet is the likely source of *Salmonella* [41]. In some instances, the dogs may not be colonised by *Salmonella* and may just be passive carriers in which food-borne *Salmonella* is transiently passing through the intestines. However, studies have shown that raw meat diets contaminated with *Salmonella* can lead to abortions and high levels of morbidity and mortality in greyhounds through *Salmonella* infection [42, 43]. In contrast to other studies, none of the retired greyhounds in this study tested positive for *Salmonella* and this may be due to the fact that they were all fed commercial diets.

In recent years there has been a trend towards the feeding of commercially manufactured pet food in many countries including the UK [9]. This is consistent with the results of the accompanying questionnaire from this study, from which it was clear that the vast majority of dogs (93 % of all dogs sampled) were fed a commercial diet. This may be one of the reasons the overall prevalence of *Salmonella* in faeces from dogs in the five housing environments studied was relatively low. Although there have been reported instances of *Salmonella* outbreaks in dogs and humans that were shown to originate from commercial food sources [44], commercial pet foods are typically manufactured in such a way as to minimise the risk of contamination from *Salmonella* [45].

In the context of racing greyhounds, dogs fed raw meat diets may be at an increased risk of *Salmonella* exposure, a possibility supported by previous research. For example, in one study *Salmonella* was isolated from 80 % of the raw meat diet samples and from 30 % of the stool samples from dogs fed the diet [46]. Other studies have also demonstrated high levels of *Salmonella* in faeces from animals fed raw meat and offal diets [47]. Unfortunately, due to the overwhelming popularity of commercial dog foods used to feed dogs in the current study it is not possible to address this issue directly. This study showed that only two dogs (0.4 %) were fed a raw meat diet and neither of these dogs was positive for *Salmonella* shedding.

The cross-sectional nature of this investigation meant that only a single faecal sample was analysed from each dog, which may be the reason that the prevalence of *Salmonella* was low. However, although the prevalence of *Salmonella* might have been higher if more than one faecal culture was performed on each dog this study suggests that the exposure risk is low; however, *Salmonella* was still identified. The limitations of single faecal cultures for the isolation of *Salmonella*, due to intermittent shedding, are well documented. Dogs with experimentally-induced latent infection shed the agent irregularly for the subsequent 3–4 weeks. In rare cases this shedding continues for up to 100 days [48, 49]. Since the agent is being shed at intervals, sampling times are very important when searching the carrier status of the dogs and in the present study we can only conclude that the dogs were positive or negative for the presence of *Salmonella* at the time of sampling. As an illustration of the intermittent shedding, the *Salmonella* positive dog in this study was repeat-sampled over three consecutive days but all subsequent samples were negative for the presence of *Salmonella.*

Further to the point of variability, the *Salmonella* isolated from the household dog that tested positive was *Salmonella arizonae,* which was only isolated following pre-enrichment of the faecal sample, suggesting the microorganism was present in low numbers. This subspecies is predominately found in reptiles, such as snakes, lizards and terrapins but has also been reported in dogs [50]. In a report by Public Health England it ranked eleventh of the most frequently reported serotypes in 2013 [1]. *Salmonella arizonae* has also been reported to cause salmonellosis in humans that have consumed contaminated snake meat or ingested medicines with traces of snake [51, 52]. Typically human cases are from vulnerable groups including immunocompromised, elderly or very young children [1]. However it is the serotypes Enteritidis and Typhimurium that are amongst the most frequently isolated serotypes of *Salmonella* from clinical cases, both human and animal, in the UK [1], and so the presence of *Salmonella arizonae* in the dog in this study may be considered atypical.

## Conclusions

In conclusion, this study indicates that the prevalence of subclinical shedding of *Salmonella* in dogs from the Midlands region of the United Kingdom is low. Good hygiene, sanitation procedures, or time of sampling are likely among the reasons for the absence of *Salmonella* in the faeces of dogs investigated in this study. It could also be that these dogs are representative of the trend in many developed nations toward feeding commercially prepared dog food rather than table scraps or other foods where *Salmonella* may be more likely to be present. Clearly, further research on this subject is needed to elucidate these possibilities.

Regardless of these findings, the possibility that dogs may harbour *Salmonella* and other zoonotic pathogens should not be ignored. Public awareness of good hygiene practices, such as frequent hand washing, can help mitigate the risk of *Salmonella* infections contracted from dogs just as from other sources.

## Methods

### Sample population

Faecal samples were obtained from 436 dogs located in the Midland region of the UK between 2009 and 2012. The dogs were from different housing environments including households (*n* = 126), rescue centres (*n* = 96), boarding kennels (*n* = 43), retired greyhound kennels (*n* = 39) and a pet nutrition facility (*n* = 132). Owners volunteered to participate in the study. There were no exclusion criteria set for this study and therefore all samples collected were used. Faecal samples from each dog were accompanied by a questionnaire to determine age, sex, breed, diet, medication, gastrointestinal problems and scavenging habits.

The study was considered by the WALTHAM Ethical Review Body and Aston University Ethics committee and approval was obtained. Informed owner consent was obtained for all the dogs that participated in this study.

### Faecal sample processing

Prior to sample testing a positive control for the isolation method was prepared from a sample of fresh homogenised faeces previously determined to be negative for the presence of *Salmonella* by direct and enrichment culturing methods [47, 53, 54]. *Salmonella* Typhimurium (NCTC 74) was grown in nutrient broth (NB; Oxoid, UK) to a concentration of 10^7^ cfu/mL, determined spectrophotometrically, and 1 mL used to spike 25 g fresh faeces to produce a positive control of approximately 10^5^ cfu/g.

Freshly voided faeces were collected by the owner in sterile bags, maintained at ambient temperature, and submitted to the investigator within 24 h. Samples were analysed for the presence of *Salmonella* using standard and enrichment culture-based method [47, 53, 54]. Samples were manually homogenised inside the collection bag and 2.5 g aliquots were taken into 22.5mls Buffered Peptone Water (BPW; Oxoid, UK) which was mixed thoroughly by agitation. Serial ten-fold dilutions were prepared from the neat faecal suspension in NB and 0.1mLs of the 10^-2,^ 10^−4^ and 10^−6^ dilutions inoculated onto Xylose lysine deoxycholate (XLD) agar, Hektoen enteric agar and Brilliance *Salmonella* agar (Oxoid, UK). The plates were incubated for 24 h at 37 °C to determine the presence of *Salmonella*. The remainder of the BPW suspension was incubated at 37 °C for 24 h. Following incubation a 0.1 mL sample was directly added to 9.9 mL of Rappaport-Vassiliadis enrichment broth (RV; Oxoid, UK) and incubated for 24 h at 42 °C. A 10 μL loop full of the incubated suspension was inoculated on the three selective agars listed above. Following incubation, plates were observed for typical *Salmonella* morphology and the identity of individual colonies confirmed using Analytical Profiling Index 20E (API; BioMérieux, France), *Salmonella* agglutination and Wellcolex® Colour *Salmonella* Rapid Latex agglutination test (Oxoid, UK) according to the manufacturer’s instructions. *Salmonella* colonies were subsequently stored on beads (Microbank™, Pro-Lab Diagnostics, Canada) at −80 °C.

### Statistical analysis

The overall prevalence, and its 95 % confidence interval, was estimated using an exact one-sample binomial test [55] using GenStat v 17.1 statistical software (VSN International Ltd).

A sample size calculation found that 100 independent samples would be needed in each population type to detect a difference of 10 % from a baseline of 1 % prevalence (the previously assumed baseline according to published literature) between population types, assuming a two sample binomial test using a 5 % significance level with at least 80 % power. In addition, it was found that with 100 samples a prevalence of 1 % could be estimated to within 2 % (i.e. a 95 % confidence interval 0, 3) which is deemed fit for purpose.
